# Supplementary material for: Are circadian amplitudes and periods correlated? A new twist in the story
Source: F1000Res. 2024 Apr 23;12:1077. Originally published 2023 Aug 31. [Version 2] doi: 10.12688/f1000research.135533.2 (PMC10526121; doi:10.12688/f1000research.135533.2)
Supplement: Peer review history

# Reviewer report: Reviewer response for version 1

**DOI:** 10.5256/f1000research.148657.r202730

**Published:** 2023-09-29

**Authors:** Jae Kim

**Affiliations:** 1 Department of Mathematical Sciences, KAIST, Daejeon, South Korea

1. The authors have conducted a study on the relationship between amplitude and period in various circadian clock models. However, it is crucial to address the novelty and distinctions of this study compared to prior work, specifically the study by Bokka et al. in 2018 ( https://doi.org/10.1049/iet-syb.2018.0015). Additionally, while the models in this manuscript are based on Hill-type repression (such as the Goodwin and Gonze models), it is important to acknowledge recent research findings that reveal different repression mechanisms in the Drosophila ( https://doi.org/10.1073/pnas.2113403119) and mammalian circadian clocks ( https://doi.org/10.1098/rsfs.2021.0084). These studies demonstrate that the use of multiple repression mechanisms, including protein sequestration, can substantially alter the oscillator dynamics ( https://doi.org/10.1049/iet-syb.2015.0090). Given these discoveries, it would be valuable to explore or discuss how different repression mechanisms impact the relationship between period and amplitude, as demonstrated by Bokka et al. in 2018.
2. In the Conclusions section on page 19, please provide a reference for 'cellular biochemical rates' as a factor influencing circadian amplitudes. For instance, it was demonstrated that faster production and degradation rates of proteins can generate circadian rhythms with higher amplitudes in the Drosophila circadian clock ( https://doi.org/10.1073/pnas.2113403119).

Is the work clearly and accurately presented and does it cite the current literature?

Partly

If applicable, is the statistical analysis and its interpretation appropriate?

Yes

Are all the source data underlying the results available to ensure full reproducibility?

Yes

Is the study design appropriate and is the work technically sound?

Yes

Are the conclusions drawn adequately supported by the results?

Yes

Are sufficient details of methods and analysis provided to allow replication by others?

Yes

Reviewer Expertise:

Circadian rhythms, mathematical modeling

I confirm that I have read this submission and believe that I have an appropriate level of expertise to confirm that it is of an acceptable scientific standard.

## References

[1] : Period-amplitude co-variation in biomolecular oscillators. *IET Syst Biol* .2018;12(4) : 10.1049/iet-syb.2018.0015 190-198 10.1049/iet-syb.2018.0015 33451181 PMC8687215

[2] : Systematic modeling-driven experiments identify distinct molecular clockworks underlying hierarchically organized pacemaker neurons. *Proc Natl Acad Sci U S A* .2022;119(8) : 10.1073/pnas.2113403119 10.1073/pnas.2113403119 35193959 PMC8872709

[3] : Combined multiple transcriptional repression mechanisms generate ultrasensitivity and oscillations. *Interface Focus* .2022;12(3) : 10.1098/rsfs.2021.0084 20210084 10.1098/rsfs.2021.0084 35450279 PMC9010851

[4] : Protein sequestration versus Hill-type repression in circadian clock models. *IET Syst Biol* .2016;10(4) : 10.1049/iet-syb.2015.0090 125-35 10.1049/iet-syb.2015.0090 27444022 PMC8687308

# Reviewer report: Reviewer response for version 1

**DOI:** 10.5256/f1000research.148657.r202731

**Published:** 2023-09-18

**Authors:** Stephanie Taylor

**Affiliations:** 1 Department of Computer Science, Colby College, Waterville, Maine, USA

In this article, the authors describe the phenomenon of twist (period-amplitude inter-dependence) using a range of differential equation oscillator models, ultimately with a view to understanding circadian clocks. The simplest models (harmonic and Duffing) are used to demonstrate the non-linearity introduces a relationship between amplitude and period. The authors then switch to limit-cycle oscillators, because limit-cycle oscillators are more appropriate for studying circadian clocks. They use two approaches to studying twist. The first, referred to as "parametric twist", computes the periods and amplitudes of oscillators with varied parameter values, creating graphs that relates period to amplitude (and thus demonstrate twist - if the correlation is positive, there is positive twist, and negative correlation indicates negative twist). This parametric twist analysis is performed using two limit cycle models, one with one feedback loop and one with more (synergistic) loops. The second, referred to as "phase space twist", examines the response of a single oscillator to a temporary change in one parameter, mimicking signaling. A separate limit-cycle model (a Poincare oscillator) is used for this analysis. Important to the discussion of phase space twist is the concept of isochrons, which are useful for understanding the effects of perturbations on the phase and speed of the clock (does it speed up or slow down the clock?) and it is easy to see how amplitude and phase response are related, when we look at how curved the isochrons are. The authors relate these analyses to phase response curves, recovery from jetlag, the range of entrainment, and temperature compensation and conclude, that although measuring amplitude and understanding relationship between amplitude and period are highly complicated, it is nonetheless important to be mindful of the role of amplitude and its correlation to period when studying clocks.

 On the one hand, I really appreciated that the authors chose models that best illustrated the points and that the paper is organized to go from simple ideas to more complex ideas. It was helpful to have the initial explanation of twist use duffing oscillator (which has non-linearity) as a contrast to a harmonic oscillator (which doesn't). It provided a nice introduction to the cause of twist and helped me to develop an intuition. Then, it made sense to use a very simple (3-ODE Goodwin oscillator) as the first example of a limit cycle oscillator. Since it has so few parameters, it was possible to perturb them one at a time and in pairs to illustrate a good portion of the possibilities for how one might observe heterogeneity in the system. Then, moving to Almeida's oscillator demonstrated how quickly the analysis can become very complex. All of this provides a nice progression of complexity. Then, we pivot a bit to the Poincare oscillator, to study twist in a more dynamical way - instead of permanently choosing different parameters, the authors model light signaling (at least, initially) and study the effects of the model returning to its limit cycle after the perturbation is over. This model is particularly appropriate for studying the dynamics of recovery because it is a 2-ode model (so we can examine it in "phase space", which is helpful for visualizing the clock) and because there is a parameter that controls how related period and amplitude are. It is also straight-forward to adjust how curved the isochrons are, which makes illustrating twist very clear. Then, after studying the response of a single oscillator to light, they use the same model to study the effects of intercellular signaling and the effects of different twist on the properties of the ensemble oscillation (e.g. how long the ensemble period is in contrast to that of individual oscillators).

 On the other hand, I would have appreciated a more explicit connection between the discussion of "parametric twist" and "phase space twist". They are both due to changes in parameter values causing changes in period and amplitude. In the one case, we measure the period and amplitude of oscillators with different (constant) parameter values. In the second case, we measure instantaneous changes in period and amplitude due to a temporary changes in parameter values (I am referring to Z(t) as a parameter here). In both cases, the shape of the isochrons in the unperturbed condition should help us to predict what will happen in the perturbed condition. There are two challenges with making that point clear with the Goodwin and Almeida models - 1) the isochrons aren't lines, they are multi-dimensional manifolds, and 2) the isochrons in the unperturbed and perturbed systems are different. So I am asking for too much to relate the two discussions by using images of isochrons, but a more explicit comparison would help the reader understand how the analyses are related to each other.

 In the analysis of the modified Poincare oscillator, the term "twist" is used differently than it is in the "parametric twist" analysis. In the parametric analysis, the twist captures the full effects of the perturbations (we plot the measured periods and amplitudes across the heterogeneous set of oscillators). In the phase space analysis, "twist" refers to a particular parameter \epsilon that, when non-zero, introduces a dependence of the speed and amplitude on each other. However, that twist parameter (\epsilon) is not the only parameter that affects the skewness of the isochrons. The relaxation rate \lambda also affects the skewness of the isochrons. And the skewness of the isochrons affects the changes in speed and amplitude when the oscillator is signaled (i.e. a parameter is temporarily perturbed). So, if we were to measure the effects on period and amplitude (as is done with figures 7G, H, and I), that reflects that values of both \lambda and \epsilon. When I was thinking about how to relate the "parametric twist" and "phase twist" analysis, I was looking for aspects of figures 3 (Gonze model), 5 (Almeida model), and 7 (poincare model) to relate and it seemed to me that that the subfigures that related the amplitude of the oscillator to its period (3C, 3D, 3E, 3F, 3G, 3H, 5B, 5B, 5E, 7G, 7H, 7I) should be the figures that I can connect. They are labeled as twist in figures 3 and 5. In Figure 7, they show an approximation of instantaneous amplitude and period (rather than steady-state amplitude and period), so it isn't a perfect 1-1 mapping, but it is still the closest that makes sense to me. And in all of these cases, the output could be labeled twist if \epsilon didn't get to claim that label. I think it would make the concept of twist clearer to the reader if it were consistent across both analyses that twist is the "effect" we can measure. My reading of the text as it is currently written is that twist is a phenomenon we can measure in "parametric twist" analyses and that twist is a parameter that causes one change in the isochrons (but not all of the changes that can happen) that ultimately leads to something we can measure. Also, labeling the parameter as twist in the Poincare oscillator makes it seem like we need to use the Poincare oscillator to perform a "phase space twist" analysis. But we aren't limited to the Poincare oscillator for such analyses.

 Specific Points

- It would be helpful to have a little more framing text that indicates the different models are used to make different points.
- I think of the "phase space twist" analysis as analysis of recovery from temporary changes to parameters and "parametric twist" as analysis of permanent differences in parameters. If that is the way you intend readers to think about it, then I would suggest changing the names to reflect those ideas more directly. Right now, the term "phase space twist" stands out as a highly specific analysis - the model most be 2D so you can draw the isochrons. But I think that is limiting. I totally embrace idea that there is both heterogeneity among oscillators and temporary changes due to intercellular signaling, so I would love to see it characterized that way more explicitly. What about "twist due to heterogeneity" and "twist due to signaling"? They aren't quite zippy enough and rely on my characterization of twist as a measurable phenomenon, so I don't push those terms exactly.
- On page 8, when you are introducing isochrons, it would be helpful to have a figure to refer to. The most natural figure is Figure 7, but that would force you to make an unnatural re-ordering of the figures. So maybe introduce a new figure?
- On Page 11, (analyses shown in Figure 3), how did you choose which parameters to pair together?
- On Page 11, there is a discussion of the Hopf bifurcation that is interesting (and highlights how sensitivity twist is to our choice of parameters). It occurs to me that this has implications for modeling SCN oscillators as they recover from TTX treatment. The individual oscillator amplitude crashes and the oscillators lose synchrony during TTX and both recover after TTX is washed out. Is there anything you could add to the discussion about this?
- On Page 12, in the paragraph about how overall twist effects depend not only on the parameter being studies, but also on the variable that is measured, I was reminded of early modeling studies that showed not-immediately intuitive results. In his 1995 fly model paper (A model of circadian oscillations in the Drosophila period protein, Proc. R. Soc. Lond. B, 1995), Goldbeter showed that the rate of (doubly-phosphorylated) PER degradation affected both period and amplitude and that increasing this rate led to PER amplitude increasing (rather than decreasing, as one might expect). This can be reasoned through, but it is another illustration of the complexity of the non-linear feedback and the relationship between period and amplitude. Is this worth bringing up here or on the discussion?
- Figure 6. Why are there no yellow dots on the limit cycle for A and B? Also, maybe make the dot color on the limit cycle different from yellow (e.g. to blue), so that you can describe what is happening over time with even more clarity (i.e. that the simulation eventually reaches the "blue" dot after enough cycles).
- Figure 8 and text referring to it. I appreciate that you relate twist to entrainment and PRCs. I think this is interesting and shows why twist is important to understand.
- Page 16 first paragraph. "This suggests that single oscillator twist might also affect how coupling synchronizes a network of oscillators" is too weak. Given that coupling (M) and the perturbation used in Figure 7 (Z(t)) both affect the model the same way (they both are added to the rate equation for the x variable), Figure 7 shows that the PRCs are affected by twist. Not only are they affected, they are affected dramatically - Figures 7K and 7L look like they are opposite each other (i.e. flipped sign). This means we are guaranteed that the oscillators will respond differently to intercellular signaling and that that the ensemble oscillation will be different.
- Page 16, in the discussion of the ensemble period, we see that for this model that positive twist leads to longer periods (and negative twist to shorter). What is missing from this discussion is that the relative phase of the signal and of the PRC of the receiver determine the period of the ensemble. Positive twist leads to longer periods because the mean field is determined by the value of the x variable. If it were determined by a variable with the opposite phase, then we would have the opposite effect. This affects the related paragraph in the Discussion as well.
- Page 17. The meaning of "For this reason we have not termed twist, in the context of conservative oscillations, parametric or phase space" wasn't clear to me. I think you mean you can't study twist in non-limit-cycle oscillators in the same way you can for limit cycle oscillators. Is that correct?

Is the work clearly and accurately presented and does it cite the current literature?

Yes

If applicable, is the statistical analysis and its interpretation appropriate?

Yes

Are all the source data underlying the results available to ensure full reproducibility?

Yes

Is the study design appropriate and is the work technically sound?

Yes

Are the conclusions drawn adequately supported by the results?

Yes

Are sufficient details of methods and analysis provided to allow replication by others?

Yes

Reviewer Expertise:

I study the response of limit cycle oscillators to signals and sensitivity analysis involving isochrons.

I confirm that I have read this submission and believe that I have an appropriate level of expertise to confirm that it is of an acceptable scientific standard.

# Reviewer report: Reviewer response for version 1

**DOI:** 10.5256/f1000research.148657.r202732

**Published:** 2023-09-18

**Authors:** Jihwan Myung

**Affiliations:** 1 Graduate Institute of Mind, Brain and Consciousness (GIMBC), Taipei Medical University, Taipei, Taiwan

In this manuscript, the authors make a comprehensive exploration of the "twist" (amplitude-period correlation) in nonlinear oscillators, especially in the context of circadian rhythms. The Introduction insightfully summarizes the background and offers a clear explanation of the topic. While some sections exhibit a degree of coarseness in their explanations, the central theme remains consistently and clearly highlighted. Overall, this manuscript is a noteworthy addition to the field, and I recommend its acceptance for indexing with minor revisions.

 Minor comments:

 1. Abstract: I suggest changing "..., fastening recovery..." to either "speeding up recovery" or "accelerating recovery".

 2. p.4, subsection "Goodwin-like models": I recommend changing "sigmoidal Hill curve" to "Hill curve", as "sigmoidal" often refers to forms involving exponential functions.

 3. p.4, subsection "Goodwin-like models": Add biological interpretations of the variables x, y, and z (mRNA, ...).

 4. p.5. ll.4-5: While the Gonze model[33] describes degradation processes with Michaelis-Menten dynamics, it may not provide sufficient historical context. I recommend citing Kurosawa & Iwasa (2002) ^1^ for a more comprehensive historical perspective.

 5. p.6, Table 2: The reference [36] should be spelled out in "The default values are taken from [36].

 6. p.11. Figure 4: It is unclear if the graphs are figurative cartoons or based on actual numerical data from Gonze oscillators.

 7. pp.11-12: The justification for the use of the Almeida model can be described. For example, why not use the detailed Gonze model or Becker-Weimann model?

 8. p.13, ll.1-2: It is challenging to assert that the "parametric twist" emerges solely due to a heterogeneous population. While it describes the average oscillator behavior in a population (often assumed to be normally distributed), it essentially characterizes the behavior of a single representative oscillator.

 9. p.14, "... 24 h, matching the intrinsic oscillator's frequency (Figure 7P).": I suggest to rephrase it to "... 24 h, matching the oscillator's intrinsic period (Figure 7P)." The change from "frequency" to "intrinsic period" would provide more specificity and clarity.

 10. p.18  "the longer period that has been observed in dispersed... , but not in dispersed SCN neurons in culture": In light of the findings in Figure 5, it's noteworthy that when Bmal1 oscillation is observed (as opposed to Per2), the SCN period lengthens under pharmacological decoupling or physical sub-sectioning of the SCN (Myung *et al., *2012 ^2^),.

 11. p.18 "Thus, a more plausible scenario may involve local coupling...": Especially in the SCN, there are papers that highlight the role of specific coupling among subregions, which can reproduce the wave-like spatial patterns and seasonal encoding of circadian time. These can be discussed.

Is the work clearly and accurately presented and does it cite the current literature?

Partly

If applicable, is the statistical analysis and its interpretation appropriate?

Yes

Are all the source data underlying the results available to ensure full reproducibility?

Yes

Is the study design appropriate and is the work technically sound?

Yes

Are the conclusions drawn adequately supported by the results?

Yes

Are sufficient details of methods and analysis provided to allow replication by others?

Yes

Reviewer Expertise:

Circadian rhythms, computational modeling of circadian clocks, suprachiasmatic nucleus (SCN), bioluminescence reporter imaging

I confirm that I have read this submission and believe that I have an appropriate level of expertise to confirm that it is of an acceptable scientific standard.

## References

[1] : Saturation of enzyme kinetics in circadian clock models. *J Biol Rhythms* .2002;17(6) : 10.1177/0748730402238239 568-77 10.1177/0748730402238239 12465890

[2] : Period coding of Bmal1 oscillators in the suprachiasmatic nucleus. *J Neurosci* .2012;32(26) : 10.1523/JNEUROSCI.5586-11.2012 8900-18 10.1523/JNEUROSCI.5586-11.2012 22745491 PMC6622328
